# Molecular Design and Functional Control of Novel Self-Oscillating Polymers

**DOI:** 10.3390/ijms11020704

**Published:** 2010-02-10

**Authors:** Yusuke Hara, Shingo Maeda, Shuji Hashimoto, Ryo Yoshida

**Affiliations:** 1 Department of Applied Physics, Waseda University, 3-4-1 Okubo Shinjuku-ku, Tokyo, 169-8555, Japan; E-Mails: maeshin@shalab.phys.waseda.ac.jp (S.M.); shuji@waseda.jp (S.H.); 2 Department of Materials Engineering, Graduate School of Engineering, The University of Tokyo, 7-3-1 Hongo, Bunkyo-ku, Tokyo, 135-8656, Japan; E-Mail: ryo@cross.t.u-tokyo.ac.jp (R.Y.); 3 PRESTO, Japan Science and Technology Agency, 4-1-8 Honcho Kawaguchi, Saitama, 332-0012, Japan

**Keywords:** self-oscillation, polymer chain, BZ reaction, gel, polymer actuator

## Abstract

If we could realize an autonomous polymer system driven under biological conditions by a tailor-made molecular design, human beings could create unprecedented biomimetic functions and materials such as heartbeats, autonomous peristaltic pumps, *etc.* In order to achieve this objective, we have investigated the molecular design of such a polymer system. As a result, we were the first to demonstrate a self-oscillating polymer system driven in a solution where only malonic acid existed, which could convert the chemical energy of the Belousov-Zhabotinsky (BZ) reaction into a change in the conformation of the polymer chain. To cause the self-oscillation in solution, we have attempted to construct a built-in system where the required BZ system substrates other than the organic acid are incorporated into the polymer itself. That is, the novel polymer chain incorporated the metal catalyst of the BZ reaction, a pH-control site and an oxidant supply site at the same time. As a result of introducing the pH control and oxidant supply sites into the conventional-type self-oscillating polymer chain, the novel polymer chain caused aggregation-disaggregation self-oscillations in the solution. We clarified that the period of the self-oscillation of the novel self-oscillating polymer chain was proportional to the concentration of the malonic acid. Therefore, the concentration of the malonic acid can be determined by measuring the period of the novel self-oscillating polymer solution. In this review, we introduce the detailed molecular design of the novel self-oscillating polymer chain and its self-oscillating behavior. Moreover, we report an autonomous self-oscillating polymer gel actuator that causes a bending-stretching motion under the constant conditions.

## 1. Introduction

Under thermodynamic equilibrium conditions, autonomous motions of macroscopic systems are tightly restricted, in the accordance with the Second Law of Thermodynamics. On the other hand, under thermodynamically open conditions, many living organisms can generate an autonomous motion without external driving stimuli. In a living body, the mechanisms generating autonomous and stimuli-responsive activities are inherent. Moreover, all living organisms involve a system capable of isothermal conversion of chemical energy into mechanical work. The biological system is significantly efficient because the chemical energy is directly converted the mechanical energy without intermediate steps. If autonomous polymer systems could be realized by completely synthetic polymers like living organisms acting under biological conditions, unprecedented biomimetic materials would be created. Here we report biomimetic polymers that cause self-oscillations induced by the Belousov-Zhabotinsky (BZ) reaction. The BZ reaction is well known for exhibiting temporal and spatiotemporal oscillating phenomena [1–9]. Until now, many researchers have studied the BZ reaction experimentally and theoretically because it can be treated as a simple model for the formation of a spatiotemporal structure, such as spiral pattern or target pattern in an unstirred solution, and multistability, periodicity, multiperiodicity in a stirred solution. The overall process of the BZ reaction is the oxidation of an organic substrate by an oxidizing agent in the presence of the catalyst under acidic conditions. In the BZ reaction, the metal catalyst undergoes spontaneous redox self-oscillations. Ruthenium tris(2,2’-bipyridine) is a metal catalyst for the BZ reaction that displays a significantly different solubility in the oxidized and reduced state. Therefore, in the BZ reaction, the metal catalyst undergoes an autonomous solubility change. By utilizing the autonomous solubility change of the Ru catalyst, we attempted to construct an autonomous self-oscillating polymer system by introducing the Ru catalyst into the polymer chain. In our previous investigations, we synthesized a polymer chain [10] and gel [11,12] composed of poly(*N*-isopropylacrylamide) [poly(NIPAAm)] covalently bonded to [ruthenium (4-vinyl-4’-methyl-2,2-bipyridine) bis(2,2’-bipyridine)bis(hexafluorophosphate), Ru(bpy)_3_] as a catalyst of the BZ reaction. As a result, we were the first to report the autonomous and spontaneous aggregation-disaggregation self-oscillation of the polymer chain and the swelling-deswelling self-oscillation for a polymer gel under the constant conditions in the presence of the BZ substrates other than the metal catalyst. However, the operating conditions of the self-oscillating polymer system are limited to a non-physiological environment where the strong acid and the oxidant coexist. For extending the application field to biomaterials, a more sophisticated molecular design to effect the self-oscillation under physiological conditions is needed. In this review, we introduce the detailed molecular design of a self-oscillating polymer system to drive the self-oscillation in a solution where only malonic acid exists. Moreover, we show an autonomous self-oscillating polymer gel actuator that causes the desired bending-stretching motion under constant conditions.

In order to operate the autonomous polymer system in the solution, we attempted to construct a built-in system where the BZ substrates other than organic acid are incorporated into the polymer chain. As the first step towards this objective, acrylamide-2-methylpropane sulfonic acid (AMPS) was incorporated into the poly(NIPAAm-*co*-Ru(bpy)_3_) chain as a pH control site [13]. Furthermore, as the second step, methacrylamidopropyltrimethylammonium chloride (MAPTAC) with a positively charged group was incorporated into the poly(NIPAAm-*co*-Ru(bpy)_3_) as a capture site for an anionic oxidizing agent (bromate ion) [14]. The bromate ion was introduced into the MAPTAC-containing polymer chain through an ion-exchange process. As a result of providing the pH-control site or the oxidant supply site into the poly(NIPAAm-*co*-Ru(bpy)_3_), we were the first to succeed in causing the aggregation-disaggregation self-oscillations of the polymer solutions under acid-free and oxidant-free conditions, respectively. Moreover, as the final step for the accomplishment of the stated purpose, we synthesized a novel polymer chain in which both the pH control and oxidant supply sites in the poly(NIPAAm-*co*-Ru(bpy)_3_) were introduced at the same time. As a result, we succeeded for the first time in causing the aggregation-disaggregation self-oscillation of the novel polymer in a solution in which only one BZ substrate (malonic acid) existed [15]. From the analysis of the self-oscillating behaviors for the novel self-oscillating solution, it was observed that the waveform and the period were significantly affected by the concentration of the malonic acid.

On the other hand, in the course of these investigations, we found that the self-oscillating polymer chain with AMPS has a high potential for causing novel self-oscillating behaviors. By utilizing the AMPS-containing polymer system, we were the first to succeed in causing an on-off switching of the transmittance self-oscillation and a viscosity self-oscillation. [16,17] Moreover, by introducing the AMPS into the conventional-type self-oscillating gel, the gel with the AMPS was modified the displacement of the swelling-deswelling self-oscillation. By utilizing the large displacement of the AMPS-containing self-oscillating gel, we were also the first to succeed in constructing gel actuators such as a self-walking gel and a matter transport gel system. [18–20] In this review, we introduced a novel autonomous bending-stretching actuator. We believe that the development of the novel self-oscillating polymer systems may lead to the construction of the novel biomimetic soft robots, and may inspire novel nonlinear experimental and theoretical considerations.

## 2. Materials and Methods

### 2.1. Polymerization

#### 2.1.1. Synthesis of poly(NIPAAm-*co*-Ru(bpy)_3_-*co*-AMPS)

Using NIPAAm (2.0 g), AMPS (7.0 g), Ru(bpy)_3_ monomer (1.0 g) and 2,2’-azobisisobutyronitrile (AIBN, 0.13 g) as an initiator, poly(NIPAAm-*co*-Ru(bpy)_3_-*co*-AMPS) was synthesized by radical polymerization in a mixture of methanol (32 g) and water (8 g) using a total monomer concentration of 20 wt%. The polymerization was carried out at 60 °C for 24 h *in vacuo*. The resulting reaction mixture was dialyzed against water for 4 days followed by methanol for 3 days, and then freeze-dried.

#### 2.1.2. Synthesis of poly(NIPAAm-*co*-Ru(bpy)_3_-*co*-MAPTAC)

Using NIPAAm (2.5 g), MAPTAC (6.5 g), Ru(bpy)_3_ monomer (1.0 g), and AIBN (0.35 g) as an initiator, poly(NIPAAm-*co*-Ru(bpy)_3_-*co*-MAPTAC) was synthesized by radical polymerization in methanol (40 g). The polymerization was carried out at 60 °C for 24 h *in vacuo*. The resulting reaction mixture was dialyzed against methanol for 3 days and then water for 4 days. For exchanging the counter ion, the polymer was dissolved in NaBrO_3_ aqueous solution (1 M) and dialyzed against pure water for 30 days with repeating exchanging the water to remove excess Na^+^ and BrO_3_^−^ ions, and then freeze-dried.

#### 2.1.3. Synthesis of poly(NIPAAm-*co*-Ru(bpy)_3_-*co*-AMPS-*co*-MAPTAC)

Poly(NIPAAm-*co*-Ru(bpy)_3_-*co*-AMPS-*co*-MAPTAC) was synthesized by radical polymerization in a methanol (31.80 g) and water (31.80 g) mixture using a 20 wt% total monomer concentration of NIPAAm (1.20 g), Ru(bpy)_3_ monomer (1.28 g), AMPS (13.05 g) and MAPTAC (0.47 g) and 2,2’-azobis(2-methylbutyronitrile)isobutyronitrile (V-59, 0.41 g) as an initiator. The polymerization was carried out at 80 °C for 24 h *in vacuo*. The resulting reaction mixture was dialyzed against methanol for 3 days and then water for 4 days. In order to exchange the counter ion, the polymer was dissolved in NaBrO_3_ (1M) and NaBr (0.5M) aqueous solution and dialyzed against pure water for 15 days with repeating exchanging the water to remove excess Na^+^, Br^−^ and BrO_3_^−^ ions. Moreover, the counter ion in the AMPS site was exchanged Na^+^ for H^+^ using ion-exchange resin, and then quickly freeze-dried.

#### 2.1.4. Synthesis of poly(NIPAAm-*co*-Ru(bpy)_3_-*co*-AMPS) gel

The poly(NIPAAm-*co*-Ru(bpy)_3_-*co*-AMPS) gel was synthesized by radical polymerization in a mixture of methanol and water (1:1 w/w) at a total monomer concentration of 20 wt%. The monomer solution including NIPAAm, AMPS, Ru(bpy)_3_ monomer, and AIBN as the initiator, was injected between a glass plate and a Teflon plate separated by silicone rubber as a spacer (thickness: 0.5 mm), and then polymerized at 60 °C for 18 h. After gelation, the gel membrane was soaked in pure methanol for a week to remove unreacted monomers. The gel was carefully hydrated by dipping it into a graded series of methanol/water mixtures, for 1 day each in 75, 50, 25, and 0% ratios.

### 2.2. LCST Measurements

The lower critical solution temperature (LCST) of the self-oscillating polymer solution was measured under the reduced and oxidized states by utilizing Ce(SO_4_)_2_ as an oxidizing agent and Ce_2_(SO_4_)_3_ as a reducing agent, respectively. The polymer solutions were prepared by dissolving the polymer chain in an aqueous solution, respectively. LCST measurements were carried out with a spectrophotometer (Shimadzu, Model UV-2,500) equipped with a thermostatic controller and magnetic stirrers. The transmittance (%) of the polymer solution at 570 nm was then recorded by raising the temperature at a rate of 0.5 °C/min.

### 2.3. Measurement of Transmittance Self-oscillations

The self-oscillating polymer solutions were prepared by dissolving the polymer in an aqueous solution. The transmittance self-oscillations for the polymer solutions were measured under constant temperature and stirring conditions. In order to detect the transmittance change which is based on the autonomous aggregation-disaggregation change, a 570-nm wavelength was used because it corresponds to the isosbestic point for the reduced and oxidized states of the Ru(bpy)_3_ in the polymer chain. The time course of the transmittance at 570 nm was monitored by a spectrophotometer.

### 2.4. Measurement of Periodic Bending and Stretching Self-oscillating Profiles for the Gel

The gel membrane (length 9.0 mm, width 2.0 mm) was immersed in 8 mL of an aqueous solution containing malonic acid ([MA] = 0.0625 M), sodium bromate ([NaBrO_3_] = 0.084 M), and nitric acid ([HNO_3_] = 0.894 M). One end of the gel strip was sandwiched in the incision of the silicone rubber. The bending and stretching oscillations for the gel strip were measured under constant temperature. Shape changes of the gel strip were observed and recorded using a microscope (Fortissimo Corp., WAT-250D), LED light (LEDR-74/40W), and a video recorder (Wooju, Time Lapse Recorder).

## 3. Results and Discussion

**Figure 1.**
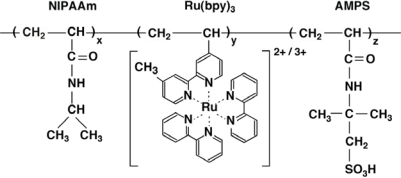
Chemical structure of poly(NIPAAm-*co*-Ru(bpy)_3_-*co*-AMPS).

**Figure 2.**
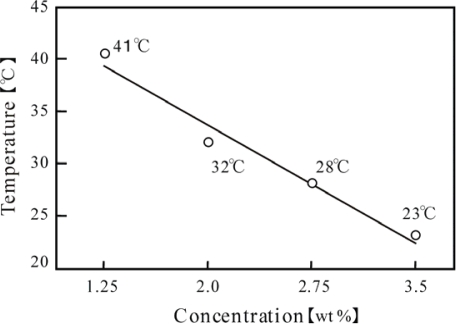
Relationship between concentration of the polymer solution and LCST for the polymer solutions in the reduced Ru(bpy)_3_^2+^ state (reprinted with permission from ref. [13]; © The American Chemical Society).

We measured the LCST (Lower Critical Solution Temperature) for different concentrations of the poly(NIPAAm-*co*-Ru(bpy)_3_-*co*-AMPS) (Figure 1) in the reduced and oxidized state, respectively. In the oxidized state, the polymer solutions have no LCST in any of the polymer concentrations (1.25, 2.0, 2.75, 3.75 wt%). This is because the polymer chain in the oxidized state contained strongly hydrophilic oxidized Ru(bpy)_3_^3+^ parts, in addition to the hydrophilic anionic charged AMPS component. These two strongly hydrophilic components in the polymer chain prevented the detachment of water molecules even at high temperatures. As a consequence, there were no LCSTs in the oxidized state. Generally, this phenomenon is observed in polyelectrolyte polymer chains which consist of the NIPAAm component. This tendency becomes remarkable with the increasing content of the ion-charged component. On the other hand, the relationship between the LCST in the reduced state and the polymer concentration is summarized in Figure 2. The LCST decreased with increasing polymer concentration. These results cannot be explained by only the general mechanism of LCST, due to the detachment of the water molecules from the polymer chains near the LCST. The polymer chain containing the reduced Ru(bpy)_3_ moiety becomes extremely hydrophobic, because the bipyridine ligands surrounding the Ru ion exert a greater influence on the solubility of the polymer chain as compared with the ionization effect of the Ru ion. In our previous study we observed that as the Ru(bpy)_3_ content increased, the sharpness of the change in transmittance with temperature became duller in the reduced state, due to the hydrophobic interaction between the polymer chains [10]. In general, hydrophobic polymer chains have a high aggregation ability originating from the hydrophobic interaction. This tendency increases with increasing the polymer concentration. Therefore, as shown in Figure 2, the LCST exhibited a linear dependence on the polymer concentration.

**Figure 3.**
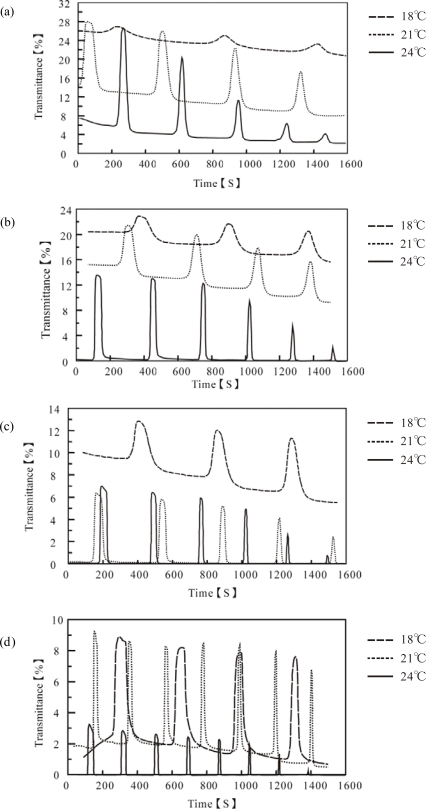
Oscillating profiles of optical transmittance for poly(NIPAAm-*co*-Ru(bby)_3_-*co*-AMPS) solutions at several constant temperatures. Polymer concentrations: (a) 1.25, (b) 2.0, (c) 2.75, and (d) 3.5 wt% (reprinted with permission from ref. [13]; © The American Chemical Society).

**Figure 4.**
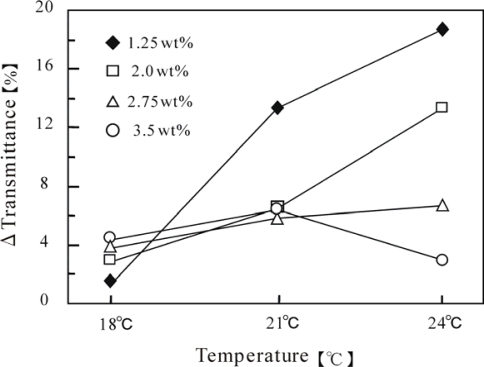
Dependence of amplitude on temperature for four polymer concentrations (1.25–3.5 wt%) (reprinted with permission from ref. [13], © The American Chemical Society).

Figure 3 shows the self-oscillating transmittance change for these polymer solutions at three constant temperatures (18, 21 and 24 °C). The aggregation-disaggregation self-oscillations have been attributed to the different solubilities of the polymer chain in the reduced and oxidized states. As shown in this Figure, the waveforms of the transmittance self-oscillation were remarkably influenced by the temperature and polymer concentration. We compared the waveforms by utilizing the oscillation amplitudes (ΔT%), which are summarized in Figure 4. As shown in Figure 4, only in the case of the 3.5 wt% concentration, the amplitude decreased at 24 °C. The LCST (23 °C) of the 3.5 wt% solution was lower than 24 °C. Since the polymer chains easily aggregated above the LCST, the aggregated polymer chains could not be easily disaggregate even in the oxidized state, which led to a decrease in amplitude. However, as the polymer concentration decreased, this tendency became less significant. The amplitudes of the polymer solutions with lower concentrations (1.25 wt% and 2.0 wt%) remarkably increased with increasing the temperature. The LCST of the 1.25 wt% and 2.0 wt% polymer solutions were 41 °C and 32 °C, respectively, which are higher than all the measured temperatures. These results demonstrate that the amplitude was greatly affected by the polymer concentration, as well as by the temperature.

**Figure 5.**
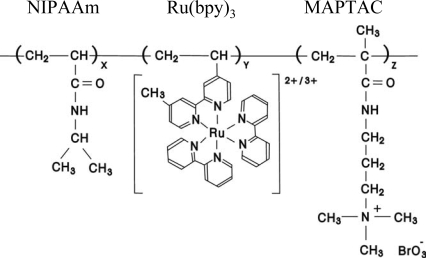
Chemical structure of poly(NIPAAm-*co*-Ru(bpy)_3_-*co*-MAPTAC).

As the second step, we synthesized the novel self-oscillating polymer chain with the cationic MAPTAC moiety as an oxidant supply site. The LCST for the poly(NIPAAm-*co*-Ru(bpy)_3_-*co*-MAPTAC) (see Figure 5) in the reduced state was 55 °C. The LCST of MAPTAC-containing polymer chain is much higher than that of the poly(NIPAAm-*co*-Ru(bpy)_3_).

**Figure 6.**
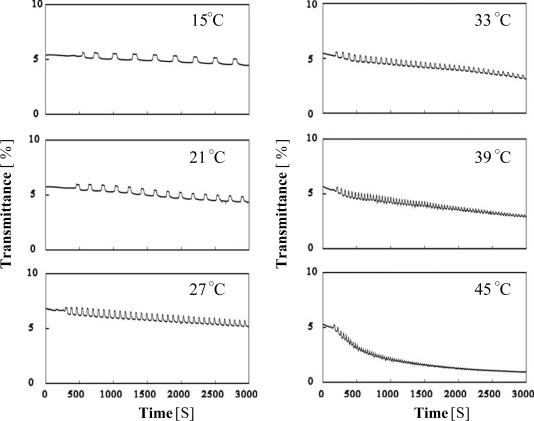
Oscillating profiles of the optical transmittance for poly(NIPAAm-*co*-Ru(bpy)_3_-*co*-MAPTAC) solutions at several constant temperatures (reprinted from ref. [14] with permission; © The American Chemical Society).

This phenomenon is generally observed in NIPAAm-based polyelectrolytes when their ionic charges increase. On the other hand, in the oxidized state, the LCST disappeared because the polymer becomes highly hydrophilic. Figure 6 shows the transmittance change of the MAPTAC-containing polymer solution at several temperatures under coexistence of only two BZ substrates (malonic acid and sulfuric acid). Since the polymer supplied the oxidizing agent (BrO_3_^−^) by itself as a counter ion to MAPTAC site, the self-oscillation was achieved without adding oxidizing agent. That is, the aggregation-disaggregation self-oscillation was caused under oxidant-free conditions. In order to cause the oscillation, a sufficient amount of BrO_3_^−^ is necessary. Therefore, the self-oscillation was not observed when the polymer concentration was below 5.0 wt%. Since the poly(NIPAAm-*co*-Ru(bpy)_3_-*co*-MAPTAC) has the LCST around 55 °C in the reduced state, the polymer has an advantage to cause self-oscillation around body temperature as shown in Figure 6.

This characteristic is of significant importance for applications to biomimetic soft robots and soft actuators such as artificial muscles, *etc.* Further, it is a special tendency for the MAPTAC-containing polymer that the self-oscillation continues for a longer time compared to the other polymers. For example, the self-oscillation lasted more than three hours at 27 °C. The longer duration of the self-oscillation is also attributed to the higher LCST of the polymer chain to avoid aggregation due to intermolecular hydrophobic interaction in the reduced state. Moreover, in contrast to poly(NIPAAm-*co*-Ru(bpy)_3_-*co*-AMPS) the polymer has no anionic site, which also acts to prevent aggregation. As shown in Figure 6, when the temperature approaches to 55 °C (the LCST in reduced state), the self-oscillation gradually damped with time. The polymer chains in the reduced state easily aggregate around the LCST due to the significant hydrophobic Ru(bpy)_3_^2+^ part. Once the polymer aggregates, they could not be easily disaggregated, even in the oxidized state. This characteristic leads to the damping aggregation-disaggregation self-oscillation.

**Figure 7.**
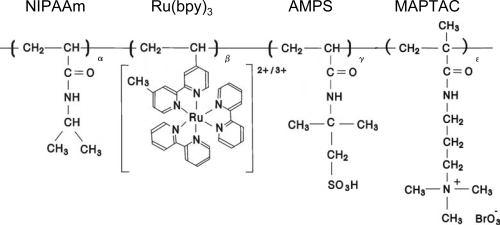
Chemical structure of poly(NIPAAm-*co*-Ru(bpy)_3_-*co*-AMPS-*co*-MAPTAC).

**Figure 8.**
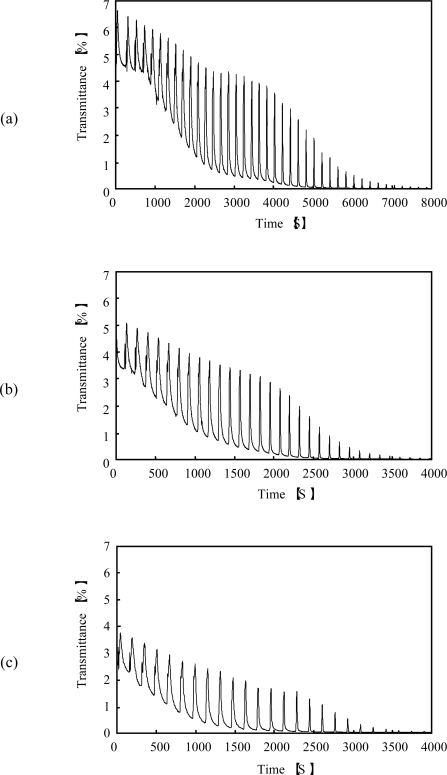
Oscillating profiles of the optical transmittance for the poly(NIPAAm-*co*-Ru(bpy)_3_-*co*-AMPS-*co*-MAPTAC) solutions at 12 °C for several concentrations of malonic acid: (a) [MA] = 0.3 M, (b) [MA] = 0.5 M, (c) [MA] = 0.7 M (reprinted from ref. [15] with permission; © The American Chemical Society).

As the final step for our purpose, we synthesized poly(NIPAAm-*co*-Ru(bpy)_3_-*co*-AMPS-*co*-MAPTAC), that is, the pH control and oxidant supply sites coexist in the polymer chain at the same time. Figure 8 shows the aggregation-disaggregation self-oscillation of the poly(NIPAAm-*co*-Ru(bpy)_3_-*co*-AMPS-*co*-MAPTAC) (see Figure 7) solutions at constant temperature under coexistence with only one bio-related BZ substrate (malonic acid).

**Figure 9.**
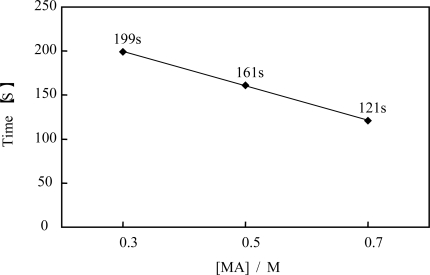
Dependence of the period on the concentration of malonic acid (reprinted from ref. [15] with permission; © The American Chemical Society).

The novel polymer chain supplied H^+^, BrO_3_^−^ and Br^−^ ions by itself as a counter ion from the AMPS, MAPTAC and Ru(bpy)_3_ sites, respectively. In order to cause self-oscillation induced by the BZ reaction under the solution where only malonic acid existed, sufficient amounts of H^+^, BrO_3_^−^ and Br^−^ ion are needed. Therefore, no self-oscillation of the novel polymer solution was observed in the concentration below 6.5 wt%. The aggregation-disaggregation self-oscillation of the novel polymer solution was attributed to the different solubilities between in the reduced and oxidized state. As shown in Figure 8, the waveforms of the transmittance were gradually dampened with time due to formation of the large polymer aggregation. The novel self-oscillating polymer chain has the betaine type polymer structure, which indicated that it has both anionic and cationic sites in the single polymer chain at same time. This structure leads to intra- and inter-molecular interactions due to the electrostatic interactions between the cationic and anionic sites in the polymer chain [21,22]. Therefore, the novel polymer solution caused damping as well as the AMPS-containing polymer solution. Figure 9 shows the oscillation periods plotted as a function of the concentration of the malonic acid. As shown in Figure 9, the period of the self-oscillation for the novel self-oscillating polymer solution was proportional to the concentration of the malonic acid. This result indicated that the concentration of the malonic acid can be measured by measuring the period of the novel self-oscillating polymer solution. By using this characteristic, the novel polymer solution can be applied as a novel sensor, which can measure the concentration of an organic acid.

**Figure 10.**
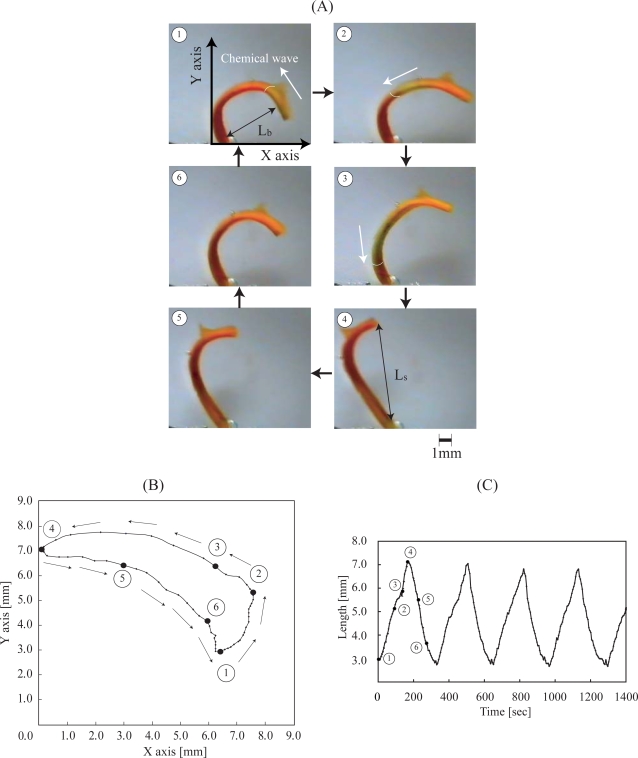
(A) Images of the repeated bending and stretching motion of the poly(NIPAAm-*co*-Ru(bpy)_3_-*co*-AMPS) gel strip in a solution of the BZ substrates ([MA] = 62.5 × 10^−3^ M, [NaBrO_3_] = 84 × 10^−3^M, [HNO_3_] = 0.894 M, 18 °C). (B) Measured motion of the tip of the gel strip. (C) Oscillating profiles of the bending-stretching motion of the gel (reproduced with permission from ref. [17]; © Wiley-VCH Verlag GmbH & Co. KGaA).

During the course process of these investigations, we found the high potential of the AMPS-containing self-oscillating polymer systems for causing novel self-oscillating behaviors. By utilizing the self-oscillating polymer system with an AMPS component, we were the first to succeed in causing viscosity self-oscillation and on-off switching of the transmittance self-oscillation. Moreover, by introducing the AMPS into the conventional-type self-oscillating polymer gel, we were the first to succeed in increasing the displacement of the self-oscillating polymer gel. Figure 10 (A) shows the periodical pendulum motion of the poly(NIPAAm-*co*-Ru(bpy)_3_-*co*-AMPS) gel strip at 18 °C. In order to drive the poly(NIPAAm-*co*-Ru(bpy)_3_-*co*-AMPS) gel strip, the gel was immersed into an aqueous solution containing all the substrates (MA, sodium bromate, and nitric acid), except the catalyst. As the solution penetrates into the gel, the BZ reaction takes place within the gel phase with the aid of the Ru(bpy)_3_ complex as the catalyst. As the reaction proceeds, the Ru(bpy)_3_ catalyst repeatedly changes between the oxidized and reduced state. First, the chemical wave evolves in the gel, and it propagates in the direction of the length at a constant speed from the tip of the gel to the edge fixed to the surface. As the chemical wave passes through the gel strip, a spontaneous bending and stretching motion of the gel occurred. While the chemical wave exists in the gel (1→4), the gel strip stretched. After that, during the reduced state (5→6), the gel bended until the next wave appeared. That is, the direction of the bending and stretching motion of the gel strip is decided by the redox Ru state. In addition, as the edge of the gel strip was fixed on the surface, the trajectory of the gel was a pendulum-like motion as shown in Figure 10 (B). The pendulum motion is induced by the propagation of the local anisotropic swelling and deswelling inside the gel. Figure 10 (C) shows the bending and stretching oscillation profiles of the whole length of the gel strip. In comparison with our previous results, the amplitude of the poly(NIPAAm-*co*-Ru(bpy)_3_-*co*-AMPS) gel is about a hundred times as large as that of the conventional-type self-oscillating poly(NIPAAm-*co*-Ru(bpy)_3_) gel.

## 4. Conclusions

We have succeeded for the first time in causing the aggregation-disaggregaion self-oscillation of a novel polymer chain in a solution where only malonic acid existed. The novel polymer chain incorporated at the same time the metal catalyst of the BZ reaction, the pH-control (AMPS) and the oxidant supply (MATPAC) sites. As for the novel quarternary polymer chain, the effect of the concentration of the malonic acid on the self-oscillating behavior was investigated. As a result, the relationship between the period and the concentration of the malonic acid was shown to be a good straight-line relation. Moreover, we succeeded in increasing the displacement of the self-oscillating gel by introducing the AMPS component into a conventional-type self-oscillating gel. By utilizing the large displacement of the gel with the AMPS, novel autonomous gel actuators were realized.
